# High-resolution micromechanical measurement in real time of forces exerted by living cells

**DOI:** 10.1080/19336918.2015.1120398

**Published:** 2015-12-08

**Authors:** Robert Swierczewski, John Hedley, Chris P. F. Redfern

**Affiliations:** a School of Mechanical and Systems Engineering, Newcastle University, Newcastle upon Tyne, United Kingdom; b Northern Institute for Cancer Research, Newcastle upon Tyne, United Kingdom

**Keywords:** cell forces, epithelial cells, fibroblasts, interferometry, uniaxial sensor

## Abstract

The aim of this study was to compare uniaxial traction forces exerted by different cell types using a novel sensor design and to test the dependence of measured forces on cytoskeletal integrity. The sensor design detects forces generated between 2 contact points by cells spanning a gap. The magnitude of these forces varied according to cell type and were dependent on cytoskeletal integrity. The response time for drug-induced cytoskeletal disruption also varied between cell types: dermal fibroblasts exerted the greatest forces and had the slowest drug response times; EBV-transformed epithelial cells also had slow cytoskeletal depolymerisation times but exerted the lowest forces overall. Conversely, lung epithelial tumor cells exerted low forces but had the fastest depolymerisation drug response. These results provide proof of principle for a new design of force-measurement sensor based on optical interferometry, an approach that can be used to study cytoskeletal dynamics in real time.

## Introduction

The integrity of multicellular organisms relies on the ability of component cells to develop and maintain adhesive and traction forces with neighboring cells or substrates. Force generation depends on physical networks of structural proteins and microtubules within cells, linking cellular components and providing the intracellular motors facilitating movement and adhesion.^1^ Measuring the force generating properties of individual non-muscle cells is a technically-difficult challenge at the interface of physics and cell biology. Early studies utilised gel-based methods to measure contractile forces exerted by non-muscle-cell populations,^2^ but the recent development of micro- and nano-fabrication techniques enables traction forces to be studied at cellular and subcellular levels.^3^ Studies have shown that capacity for force generation varies between different cell types;^4^ furthermore, disease processes can alter the biophysical properties of cells and changes in force-generation capacity may facilitate the metastasis and spread of tumor cells.^5^

Controlling force generation in cells outside their normal environment may be a clinical strategy to control the spread of tumor cells. Developing drugs or strategies to achieve this will be dependent on a better understanding of the dynamics of structural networks in relation to cell force generation. Inexpensive devices which can be used as high-throughput research tools will be important for realizing this goal. We previously reported the application of optical profilometry for cell force measurement of living cells in real time using a novel device microfabricated from silicon wafers by photolithography and plasma etching techniques.^6^ The aim of this study was use these devices to test the time-series characteristics of uniaxial force measurement in 3 different cell types representing normal epithelial cells, dermal fibroblasts and an epithelial tumor cell line, and to test the hypothesis that the forces observed are dependent on cytoskeletal integrity.

## Methods

### Cell culture

Immortalized bronchial epithelial cells 16HBE14o,^7^ hereafter HBE cells, were a gift from Monika Suwara, Newcastle University; dermal fibroblasts were primary cells cultured and passaged from human foreskin,^8^ and the A549 lung cancer cell line was a gift from Tsutomu Nobori, Mei University, Japan. Cells were cultured in Minimum Essential Medium Eagle (dermal fibroblasts; Sigma) or HyClone MEM/EBSS with Earl's balanced salts (epithelial cells; Fisher Scientific), supplemented with 10% FCS, 1% L-Glutamine and 1% Streptavidin/Penicillin solution (100 U/ml penicillin, 100 μg/ml streptomycin, Sigma). Force measurements were made in complete culture media at 37°C using HEPES-buffered medium without Phenol Red. To remove potential contaminants, such as suspended solids, from being deposited on critical parts of the sensor, all complete media were filtered using 220 nm sterile filter prior to use for sensor cell cultures. Initial seeding density in sensors was 5000–20000 cells/ml for epithelial cell lines and 20000–50000 for fibroblasts; these amounts represent a balance between incubation time and the manageability of cell migration. Culture media were changed every 3 days. Colchicine and Cytochalasin D (0.5 μmol/ml and 0.25 μmol/ml, respectively) were added to cells growing on sensors to test the dependence of force measurements on the cell cytoskeleton; optimal doses to depolymerize microtubules (colchicine) and actin filaments (Cytochalasin D) within 30 min were established empirically, using previous reports^9-12^ as a guide.

### Force sensors

**Figure 1.**
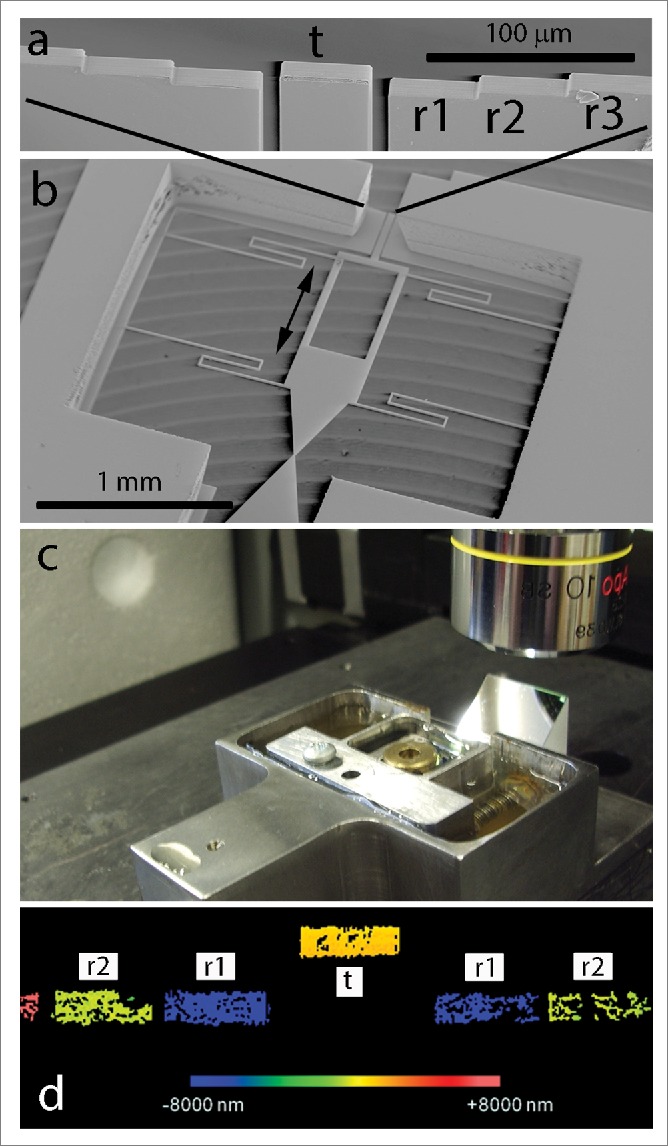
Scanning electron micrographs of a sensor (a, b), the profiler configuration with respect to the sensor holding apparatus (c) and a profile image to demonstrate the measurement principle. The test (t) and reference surfaces (r1, r2, r3) are shown in higher magnification in (a). The rest of the sensor is shown at lower magnification in (b), where the double-headed arrow indicates the direction of displacement. In (d), a color representation of reference and test surface depths (as visualised in a plane perpendicular to the direction of displacement) is shown: a pair of reference surfaces (r2) defines the reference plane; a second reference plane (r1) allows for compensation of the apparent depth effect due to the viscosity of the medium (reference surface separation or step height is 10 µm). Displacement of the test surface (t) with respect to the reference plane indicates the force present.

Details of sensor design and manufacture have been reported previously.^6^ Briefly, the force sensors were microfabricated from silicon wafers by conventional deposition, photolithography and plasma etching techniques.^6^ The sensors consisted of a 4.5 μl reservoir, in which cells were placed, leading via a narrow tip to a rigid, deflectable platform held in place with flexible ligaments. The tip had a point width of 10 μm and was separated by a gap of 2 μm from a similar 10 μm wide tip on the deflectable platform. The tip width helps limit tip occupancy to a single cell. To measure platform translation induced by a cell spanning the gap from tip to tip, test (part of the deflectable platform) and reference surfaces at the platform cross-section allowed high resolution optical profiling using interferometry for displacement measurement (Fig. 1). Silicon surfaces were functionalised for cell attachment by coating with collagen-I^6^ and the devices were reused after cleaning with 10% hydrogen peroxide in concentrated sulphuric acid (30:70), extensive washing in deionised water and sterilisation with 70% ethanol. Trypsinised cells were seeded into the reservoir in complete culture medium, using a sterile cover slip and surface tension to confine cells to the loading chamber, and grown for 3–15 days until the sensor gap was breached by a single cell. To facilitate cell tracing, cells were viably stained either by baculovirus transduction of b-actin-RFP (Cellular Lights™ Actin-RFP - Life Technologies) or with Rhodamine 6G (green) and Rhodamine B (red) (all Sigma Aldrich). Cell nuclei were stained with Hoechst 34580 (blue). Staining methods were adapted from Nosyk et al.^13^ Samples were visualised using Zeiss AxioPhot2, Nikon-Eclipse TE-2000-U epifluorescence and Zeiss LSM 700 confocal microscopes.

Forces were measured, mostly for a duration of 60 min, when single cells were observed spanning the sensor gap. Measurements were of ‘push’ forces resulting in the test surface standing proud of the reference surfaces; ‘pull’ forces are limited by the gap width and the possibility of the cell growing within the gap and are therefore less accurate. Readings were taken using a Zygo NewView 5020 profilometer at 60 s intervals using an automatic script to control data-acquisition; a 30 Hz CCD frame rate and scan depth of 40 μm took 45 s to complete, leaving 15 s to compute, save data and prepare the scanner for another acquisition cycle. An increased reading frequency of 15 s was used for an initial experiment but resulted in gaps in data acquisition due to limitations in computer memory and data transfer speeds. Therefore, for the majority of experiments, a 60 s interval between readings was used as this gave enough time to compute the 415 million acquired pixels at each step with serial transfer to the data matrix on a local hard drive. Sensor measurement stability was measured in water without cells using a sample of 6 devices over a 30 minute period each. Variations in apparent test-surface displacement in water will be due to thermal drift, environmental vibrations and instrument error. Sensor design was calibrated by measurements of sensor force-displacement curves using a JPK Nanowizard 3 Atomic Force Microscope (AFM)^6^ and a value of 10.5 Nm^−1^ for system stiffness was used to convert displacement measurements to force; this is similar to the value of 8.85 Nm^−1^ modeled from finite element analysis of ligament dimensions and physical properties.

### Data analysis

Statistical analyses and graphics were done with R.^14^ Forces generated by different cell types before the addition of colchicine and cytochalasin D were analyzed using a mixed effect linear model in the R package ‘lme4’^15^ with sensor as a random effect and test probabilities estimated with the package ‘afex’.^16^ Four-parameter logistic (Boltzmann) curves were fitted to the data from the time of colchicine and cytochalasin D addition using the package ‘drc’;^17^ for 2 of the 6 HBE experiments, the decline in force generation was too close to the end of the culture period for the lower asymptote to be estimated accurately and the ranges of the lower asymptote for 4 HBE experiments were used as constraints in the models for the other 2. Model parameters were compared between cell types by ANOVA (function ‘aov’) with TukeyHSD for pairwise comparisons; in addition, because of model-fitting constraints for 2 of the HBE experiments, key results with respect to the midpoint parameter were also checked using the non-parametric Kruskal-Wallis test with pairwise comparisons using Tukey and Kramer (Nemenyi) test (R package ‘PMCMR’^18^). Permutation entropy values^19^ from time-series force data were calculated using ‘statcomp’^20^ with bootstrapping (sample size: 1000) for confidence intervals using ‘boot’,^21^ and compared with simulated data generated randomly from a normal distribution.

## Results

### Steady-state traction forces

The aim of this present study was to compare, using a novel sensor design,^6^ the forces exerted by different cell types and to test the dependence of measured forces on cytoskeletal integrity. In initial experiments with dermal fibroblasts (DF), we recorded forces at 15 sec intervals over a period of 90 min. However, the data acquisition rate exceeded computer transfer speed and buffer storage, resulting in occasional gaps between runs of data. Nevertheless, sufficient high-resolution data were accumulated between gaps to ask whether the time-dependent variation in force exerted by the cell was due to random noise or low-dimensional chaos.^22,23^ Permutation entropy (PE)^19^ was calculated for the steady state data (mean force ± standard deviation [sd]: 2782 ± 190 nN) and compared with data simulated randomly from a normal distribution. PE values and bootstrapped 95% confidence intervals were similar for the experimental (0.74; 95% confidence range 0.704 – 0.745) and simulated (0.73; 95% range 0.705 – 0.744) data. The time-dependent variation in force generated by cells (sd 190 nN) exceeded that measured with empty sensors in water (mean sd: 44.7 nN); therefore, this analysis suggests that, over the current timescale, variation in forces exerted by cells was random rather than a result of chaotic cellular processes.

**Figure 2.**
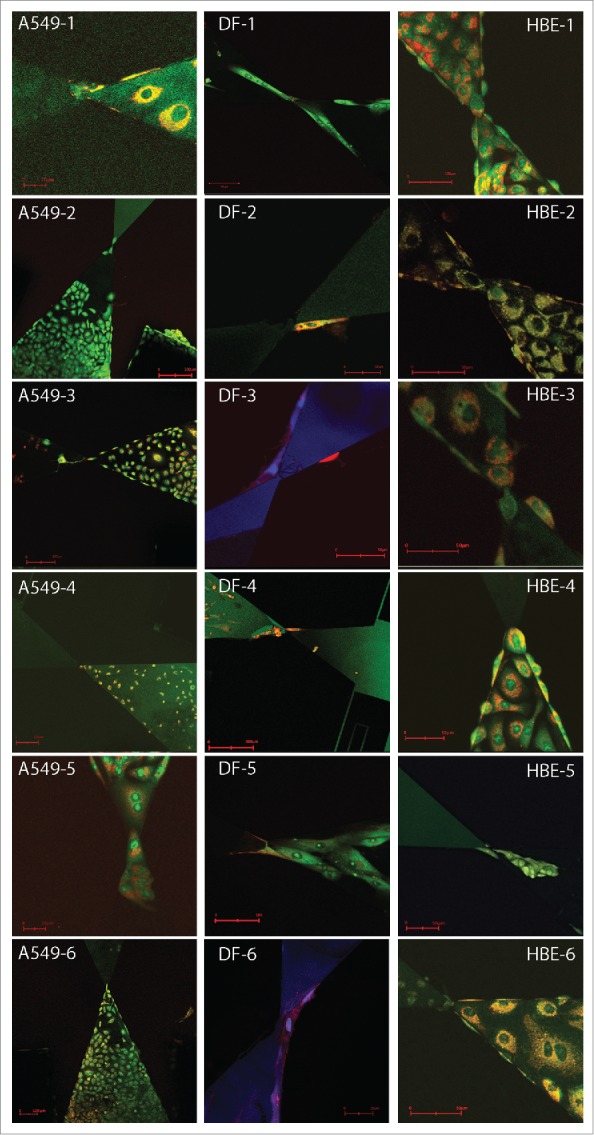
Confocal images of A549, dermal fibroblasts (DF) and HBE cells crossing the sensor gap. Scale bars (red) vary in each image: for A549 cells are 20 μm (A549-1 and -5), 100 μm (A549-3, -4 and -6) or 200 μm (A549-2); for DF cells these are 20 μm (DF-6), 50 μm (DF-2 and -3), 100 μm (DF-1 and -5) or 200 μm (DF-4); for HBE cells these are 50 μm for all except HBE-1 (100 μm).

**Figure 3.**
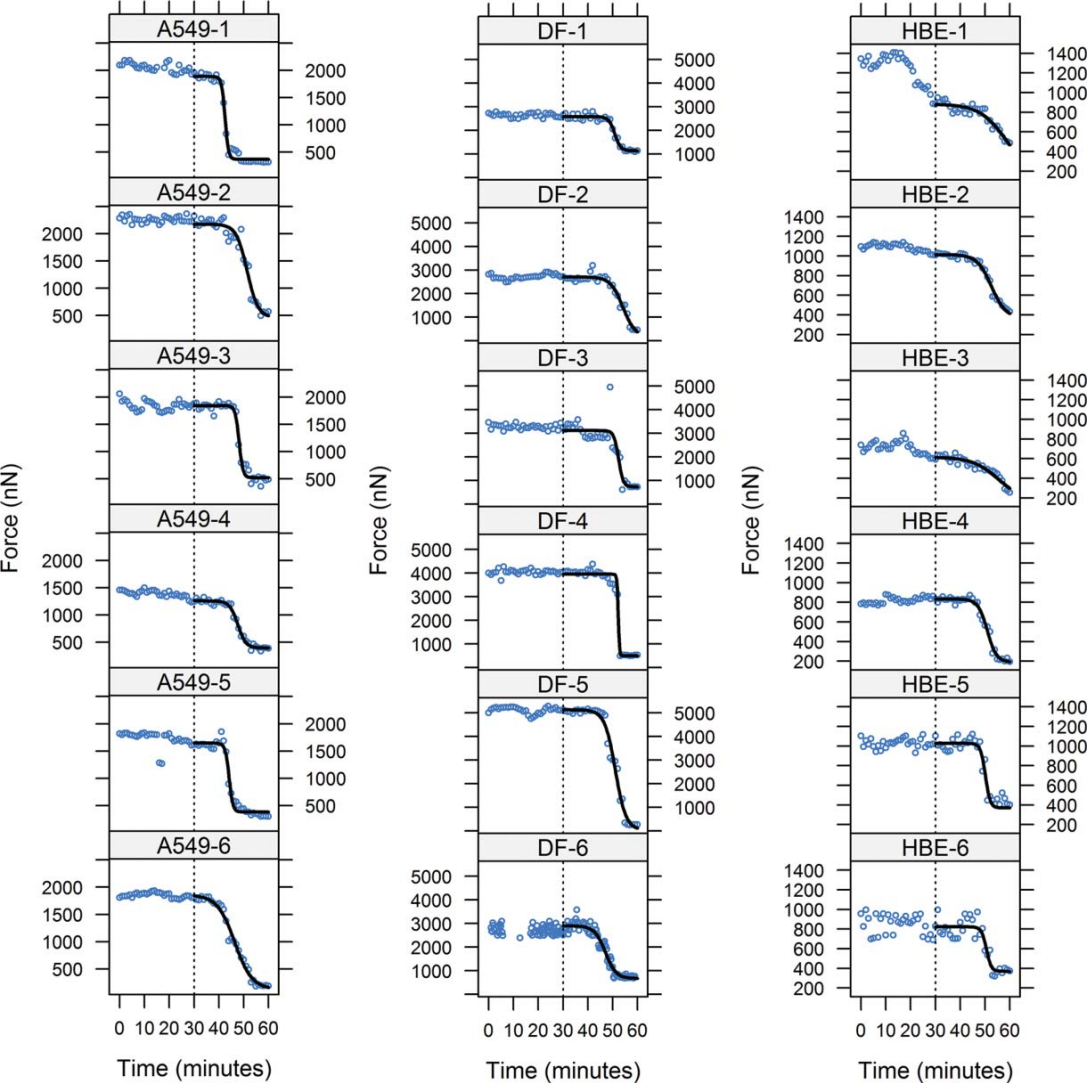
Sensor data for A549 cells (left panel), dermal fibroblasts DF, center panel) and HBE cells (right panel): blue circles are forces (nN) measured at 1 min intervals; depolymerising drugs were added at 30 minutes. The black lines from 30 min (the time of drug addition indicated by a vertical dotted line) on each plot are 4-parameter logistic curves (Boltzmann) fitted to the data. Note DF-6 was cultured for 90 min with recording at 15 s intervals: the first 30 min have been omitted for comparison with the rest of the data. Note that the vertical axis scales differ for each cell type.

**Figure 4.**
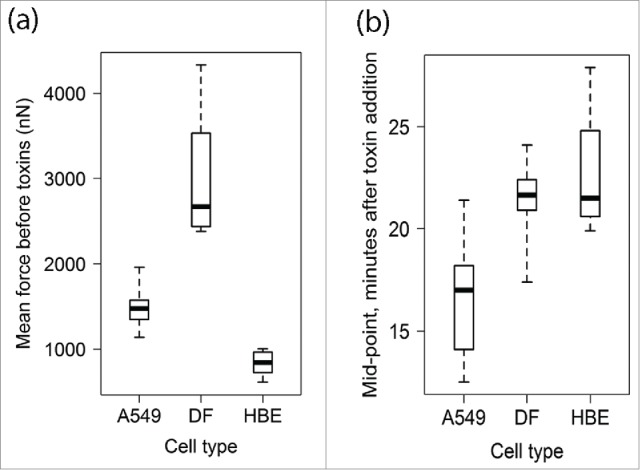
Box & Whisker plots (whiskers are minimum to maximum; boxes span the 25th to 75th percentile with the bold horizontal line representing the median) for (a) steady-state force measurements ( in nN) over the first 30 min of culture, and (b) midpoints of 4-parameter logistic curves describing the decay in force generation after addition of colchicine and cytochalasin D.

For comparisons between cells and different cell types, forces were recorded at 1 min intervals to ensure consistent data acquisition. All measurements were obtained from sensors which appeared, from fluorescence microscopy just before measurement, to have a single cell visible across the platform gap (Fig. 2); a post-hoc assessment of these images suggests that the initial assessment was correct, except perhaps in the case of DF-5 where the contribution of an additional cell cannot be excluded and may explain the higher force levels generated in this sensor experiment (Fig. 3). Sensors were held in the optical profiler for a period of 30 minutes before adding a mixture of the cytoskeletal depolymerising drugs colchicine and cytochalasin D and data acquisition continued for a further 30 min. The ‘steady-state’ forces during the 30 min before addition of depolymerising drugs, differed between cell types (linear mixed-effects analysis on log10 values by sensor, effect of cell type F_2,15_ = 51.66, P < 0.0001) and were highest with DF (mean 3004 nN) with the epithelial cell lines showing lower mean force levels (1497 and 833 nN for A549 and HBE cells, respectively; Fig. 4a); all pairwise comparisons for mean force by cell type were statistically significant (Tukey multiple comparisons, P < 0.001). For half of the HBE and A549 cultures there was some evidence for a longer-range non-random variation both increases and decreases, in force measurements within this initial culture period, and this was particularly marked for one of the HBE cultures; for all dermal fibroblasts measured, forces remained relatively steady during this period (Fig. 3).

### Force and cytoskeletal integrity

After adding colchicine and cytochalasin D, forces exerted by cells declined (Fig. 3). To compare the characteristics of force decay between cell types, 4-parameter logistic (Boltzmann) curves were fitted to the data for each experiment. In most cases, these curves provided a good fit to the data (Fig. 3) and the mean curves parameters for each cell type are summarised in Table 1. The midpoint and slope parameters (Table 1) of the symmetrical curves for each cell type were compared as objective measures of the time course of force decay. The midpoints differed significantly between cell types (ANOVA, effect of cell type F_2,15_=7.31, P = 0.006; Kruskal-Wallis test, P = 0.0124) with A549 cells reaching the midpoint sooner than HBE cells (Tukey's HSD test for pairwise comparisons: P < 0.01; non-parametric Nemenyi, P = 0.068) and dermal fibroblasts (Tukey's HSD, P < 0.05; Nemenyi, P = 0.014; Fig. 4b). The upper asymptotes reflect forces exerted at around the time of drug addition; these differed significantly between cell types as with the steady state force levels (Tukey's HSD, P < 0.05). Conversely, there was no significant difference between cell types in the rates of decline around the midpoints (ANOVA on slope parameter, effect of cell type: F_2,15_ = 0.75, P = 0.5; means for A549, DF and HBE cells were 0.9 ± 0.52, 1.37 ± 1.75 and 0.6 ± 0.45, respectively) and no cell-type specific variation in the lower asymptote or residual force (cell-type effect F_2,15_ = 1.49, P > 0.25; means ± sd for A549, DF and HBE cells were 317.0 ± 113.3, 461.1± 335.1 and 254.3 ± 103.2 nN, respectively). There was no correlation between the slope and midpoint parameters, either for all cells or within cell types. The residual forces had significantly decreased variance compared to steady-state before treatment (2-sample variance ratio test, P < 0.0001), with a mean standard deviation (34.7 nN), which was comparable in magnitude to the error of devices without cells.

**Table 1.** Parameters by cell type for the Boltzmann curves fitted to the force-decay curves.

Parameter	Slope (at midpoint)		Lower asymptote (nN)		Upper asymptote (nN)		Midpoint (minutes)	
	mean	sd	mean	sd	mean	sd	mean	sd
Cell type								
A549	0.91	0.52	317.03	113.34	1497.08	257.74	46.7	3.15
DF	1.37	1.75	461.1	335.15	2843.42	836.39	56.33	10.37
HBE	0.61	0.45	254.32	103.17	727.75	125.97	52.69	3.09

## Discussion

The devices used for this study measure linear, unidirectional forces of an intact, living cell constrained by the surface attachment dimensions and properties of the substrate platform. The data provide proof of principle that optical profilometry measurements of platform displacement relate to the biomechanical properties of individual living cells. Four main sources of force variation were apparent: (1) random fluctuations at small temporal scales which could not be assigned to low-dimensional chaotic cellular processes; (2) longer-range, time-dependent variation in ‘steady-state’ forces exerted by some cells; (3) variation in mean steady-state force levels within a cell type; (4) variation in force levels between cell types.

Many recent studies of forces exerted by cells have used micropillar devices, gels or bead-tracking systems which can measure the distribution of traction forces across a cell in relation to substrate and adhesion points,^3,24-27^ and how these relate, for example, to epithelial sheet integrity.^28^ The subcellular force resolution of such studies make direct comparisons with whole-cell, uniaxial measurements difficult. Nevertheless, such studies emphasize the role of 2 key components in biomechanics: the adhesion or contact points between cell and substrate, and the cytoskeletal structures linking these with the rest of the cell. Using drugs to depolymerise actin (Cytochalasin D) and microtubules (colchicine), demonstrates the critical role of the cytoskeleton for force transmission,^1,24,29^ and validates the force measurements using these devices. An additional consequence of actin and microtubule depolymerisation in the present study was a reduction in variance of force measurements to that of sensors without growing cells. This indicates that the high variance of force measurements with viable cells spanning the device gap was due to active intracellular processes; these processes appeared to be random in nature rather than driven by chaotic changes in a few cellular or cytoskeletal parameters.

The variations in response time (here defined by the midpoint of the response curve) emphasize differences between cell types with respect to both force generation and drug-response properties. Dermal fibroblasts exerted the greatest forces and had the slowest drug response times (or greater resistance). HBE bronchial epithelial cells, an EBV-transformed ‘normal’ cell line, had similarly-slow cytoskeletal depolymerising times but exerted the lowest forces overall. Conversely, the lung epithelial tumor cells, A549, exerted low forces but had the fastest depolymerisation drug response. For all 3 cell types, the measured force levels were comparable to those reported for other studies of forces at a whole-cell level,^4^ where fibroblasts exert forces within the range 500–5000 nN and epithelial tumor cells 300–750 nN).^30^ Although we have only studied 3 cell types, the results for A549 cells in comparison with the transformed bronchial epithelial cells are compatible with the concept that changes in biomechanical properties can occur during tumorigenesis.^31^

Forces exerted across the gap sensor will be dependent on the number of contact or adhesion points on each side and, therefore, represent traction forces in a linear dimension constrained with respect to distribution of adhesion points between the 2 device tips. Device or substrate geometry and adhesiveness may also influence cytoskeletal networks and capacity for force generation.^32^ Experiments with micropillar devices have shown that traction forces increase linearly with micropillar stiffness,^33^ but there is likely to be a limit at which adhesion breaks down; therefore, cells may have feedback mechanisms to maintain substrate contact by limiting force generation at individual contact points. As the number of adhesion points each side of the gap may vary as a result of movements of the cell during the one-hour measurement time, this may be a source of the time-dependent (non-random) variation in steady-state force levels seen in some of the experiments. Variation in the number of adhesion points may also be a factor underlying the variation in force generation between cell types. Furthermore, if force generation is regulated by feedback mechanisms from adhesion points, forces will also vary according to the nature of the substrate or cell-substrate interactions.

Variation between cell types in the capacity to generate mono-axial force and sensitivity to actin and microtubule depolymerising drugs may be due to cell-type specific differences in actin cytoskeletal organization and abundance, and to differences in cell size and shape. The data from this study indicate that force generation capacity and sensitivity to cytoskeletal disruption are separable cellular characteristics. Given the variability between cells with respect to these properties, force measurements might be utilised as a tool for investigating cytoskeletal integrity and drug sensitivity in a range of diseases, particularly cancer and diseases resulting from mutations in cytoskeletal components.^34^ With the different approaches available, it is important to consider whether mono-axial traction, as measured here, or multiaxial traction as measured by various bead or micropillar devices is the most appropriate tool. The device used here has the potential to be redesigned to deliver individual cells to an array of measuring channels. Perhaps the biggest challenge is to obtain sufficient measurement resolution and this study provides proof of principle for interferometry-based measurement which has good sensitivity and force resolution.^35,36^ To develop this approach into a practical low-cost research or high-throughput device is likely to require integration of a fiber optic interferometric platform with a multichannel culture device.

## Conclusion

The magnitude of uniaxial cell forces detected with this sensor design varied according to cell type and were dependent on cytoskeletal integrity. The response time for drug-induced cytoskeletal disruption also varied between cell types. These results provide proof of principle for a design of force-measurement sensor based on optical interferometry, an approach that can be used to study cytoskeletal dynamics in real time.

## Abbreviations

*ANOVA*: analysis of variance
*CCD*: Charge-coupled Device
*EBV*: Epstein-Barr Virus
*HBE*: immortalized human bronchial epithelial cells
*RFP*: Red fluorescent protein
*sd*: standard deviation
